# Unsupervised Wildfire Detection Using Multispectral MTG-FCI Data: A Feasibility Study

**DOI:** 10.3390/jimaging12060229

**Published:** 2026-05-27

**Authors:** Alessandro Mercatini, Nazario Tartaglione

**Affiliations:** Italian Institute for Environmental Protection and Research (ISPRA), Via Vitaliano Brancati 48, 00144 Roma, Italy; nazario.tartaglione@isprambiente.it

**Keywords:** MTG-FCI, unsupervised learning, anomaly detection, deep learning, fire detection, geostationary satellites, near-real-time monitoring

## Abstract

The launch of the Flexible Combined Imager (FCI) sensor aboard the Meteosat Third Generation (MTG) satellite enables higher temporal and spatial resolution for geostationary environmental monitoring. This study explores the feasibility of near-real-time fire detection using MTG-FCI data. Two unsupervised approaches are evaluated on data covering the Italian territory: a conventional threshold method, applying fixed radiometric thresholds and brightness temperature differences between 3.8 μm and 10.5 μm, and an experimental Lightweight U-Net autoencoder for anomaly detection. The autoencoder is trained exclusively on fire-free imagery, with fires identified as statistical anomalies in the reconstruction error, refined through local and global z-score analysis. Validation combines high-resolution Sentinel-2 imagery, Fire Radiative Power (FRP) and data from European Forest Fire Information System (EFFIS). Results demonstrate that MTG-FCI can trigger active fire alerts prior to polar overpasses in 67.32% of the synchronized cases, providing a median early detection lead time of 21.00 min and reaching an advance of up to approximately 6 h in exceptional instances. While the spatial resolution limits detailed fire-front mapping, the high temporal resolution enables a robust near-real-time alerting system, providing enhanced detection of transient fire events that are not captured by lower-frequency polar-orbiting sensors.

## 1. Introduction

Wildfires are a recurring problem in many areas that experience dry seasons, but they have become a particularly serious concern in the Mediterranean region. Vast tracts of land often experience devastating impacts each summer. The repercussions extend beyond the immediate destruction of the plant life. These fires can destabilize ecosystems for extended periods and degrade air quality, all while posing a threat to both people and property [1]. Southern European nations are especially vulnerable, where extended heatwaves and limited summer precipitation accelerate fuel desiccation, making vegetation highly flammable. In addition, shifts in social and economic practices have gradually altered the land’s use and the overall landscape. The abandonment of rural land has resulted in a buildup of unmanaged plant material, and the growth of urban areas has pushed residential zones and transportation routes near forested areas, creating conditions that favor ignition and rapid fire spread [2].

According to the European Commission’s Joint Research Centre (JRC) reports, Italy, along with Spain and Greece, is consistently among the most affected Mediterranean countries, with burned areas exceeding tens of thousands of hectares on average each year [3]. Large forest fires in Sicily, Sardinia, Calabria, and central Italy have serious socioeconomic and ecological impacts, including soil erosion, habitat loss, air pollution, and threats to human settlements and infrastructure [4].

Rising temperatures and decreasing precipitation are expected to lengthen fire seasons, resulting in more frequent and larger wildfires [5,6].

Satellite-based remote sensing has become central to wildfire monitoring at regional and global scales. Multispectral and thermal data are routinely used to detect active fires, estimate Fire Radiative Power (FRP), and map burned areas [7,8]. However, satellite monitoring is constrained by the trade-off between spatial and temporal resolution. While high spatial resolution is needed to identify small fires in complex landscapes, insufficient temporal frequency remains the main obstacle for both early detection and the tracking of rapidly evolving events.

Satellite platforms can be broadly classified into Low Earth Orbit (LEO) polar-orbiting systems and Geostationary (GEO) systems. Instruments like the Moderate-Resolution Imaging Spectroradiometer (MODIS) and Visible Infrared Imaging Radiometer Suite (VIIRS) are excellent for spotting small fires due to their high spatial resolution, reaching 375 m in the case of VIIRS [9]. However, their orbit is a major constraint: with only one or two overpasses per day, they lack the continuity needed to catch fast-moving fires. In Mediterranean regions, where fire dynamics are rapid, this temporal gap can delay detection by several hours. GEO satellites provide the continuous coverage needed to fill these temporal gaps.

Experiences of wildfire tracking using GEO satellites have been reported in a few works [10,11,12,13], especially for tracking large wildfires [14]. GEO satellites may be very effective in wildfire detection. For example, GOES detections of the wildfires are earlier than those of the VIIRS active fire products in most of the events analyzed by Zhao and Ban [15].

The SEVIRI sensor, on the Meteosat second generation (MSG) satellite, produces imagery every 15 min for near-real-time (NRT) monitoring. However, its 3 km spatial resolution at nadir remains a major drawback; such coarse data often fails to detect small or early-stage fires, particularly in the complex topography of the Mediterranean [16].

The Meteosat Third Generation (MTG) satellite, carrying the Flexible Combined Imager (FCI), improves upon previous geostationary capabilities in geostationary monitoring [17,18]. Following its 2022 launch, the sensor entered full operational service in late 2024. The FCI improves spatial resolution to 0.5–2 km and increases temporal sampling to 2.5–10 min, effectively narrowing the gap between geostationary and polar-orbiting capabilities [19]. FCI can be effectively used to retrieve FRP signals [13].

Despite the high potential of MTG-FCI for active fire detection, its operational use remains limited [20]. The sensor became operational only recently, and most studies so far have adapted threshold-based, contextual, or machine learning methods originally developed for other satellites, such as MSG-SEVIRI or polar-orbiting sensors [21,22].

Given the novelty of the MTG-FCI sensor, its operational exploitation for wildfire monitoring is still in its early stages. The objective of this study is to demonstrate that, despite the inherently limited spatial resolution of geostationary observations, the high temporal sampling frequency of MTG-FCI can be effectively leveraged for near-real-time fire detection using strictly unsupervised methods over the Italian territory. We evaluate two straightforward, lightweight approaches that do not require historical fire catalogs or manual labeling: a conventional threshold-based method and a lightweight U-Net autoencoder specifically trained to isolate thermal anomalies against a fire-free background. By testing these unsupervised workflows on the newly available MTG data stream across several documented fire events, covering different vegetation types and atmospheric conditions, this work aims to verify whether a high temporal cadence can compensate for spatial constraints, providing an accessible and computationally efficient framework for early fire alerting.

## 2. Materials and Methods

### 2.1. MTG FCI Data

The MTG-FCI Level 1-C datasets used in this feasibility study were acquired in an NRT through the EUMETCast High-Volume Service 3 (HVS-3) reception station, equipped with a dedicated antenna system installed at the ISPRA (Italian Institute for Environmental Protection and Research) headquarters in Rome. This direct reception infrastructure ensures a low-latency data flow, which is a fundamental requirement for developing and testing operational wildfire monitoring systems in a NRT framework.

The dataset is derived from a geographic crop focused on Italy, extracted from the Full Disc High Spectral Resolution Imagery (FDHSI) product (radiometrically calibrated and geometrically corrected). These include multiple visible (VIS), near-infrared (NIR), and infrared (IR) spectral channels, as detailed in the official EUMETSAT user documentation [23]. Of the 16 spectral channels provided by the MTG-FCI instrument, only 4 bands were selected, based on their characteristics (Table 1). This choice was made to balance high detection sensitivity with the computational efficiency required for a lightweight, NRT architecture. The 1.6 μm NIR, 2.2 μm NIR, and MWIR channels were selected for their established role in wildfire monitoring, particularly as core components of the standard RGB product for wildfire temperature; these bands are particularly sensitive to high-temperature anomalies and can effectively penetrate smoke-saturated environments. Finally, we included the TIR band as a stable thermal reference. Being positioned in the center of the atmospheric window, it provides a nearly direct observation of surface temperature, allowing the model to better distinguish between solar-induced ground heating and actual active combustion [24].

Although FCI is a multispectral sensor with a nominal resolution of 2 km in the IR bands, the MWIR channel allows for a sophisticated form of sub-pixel thermal analysis. This is possible thanks to the non-linear nature of the Planck function: at these shorter infrared wavelengths, the spectral radiance increases much more sharply with temperature than in the thermal infrared. Consequently, even a small wildfire occupying only a tiny fraction of the pixel area contributes disproportionately to the total radiance, effectively addressing the spatial mixing challenge typical of coarse-resolution imaging [25,26].

Infrared channels are provided as brightness temperatures (BT, in K), while NIR channels are expressed as top-of-atmosphere (TOA) bidirectional reflectance factors (%). No additional atmospheric correction was applied, as the proposed methodology relies on relative spectral and thermal contrasts between fire-affected pixels and the surrounding background.

To ensure consistency for multi-band analysis, all selected channels were resampled onto a common equispaced grid with a nominal spatial resolution of 1 km, defined in the ETRS89–LAEA coordinate reference system (EPSG:3035). A nearest-neighbor resampling approach was adopted to preserve the original radiometric properties and to avoid spectral smoothing. This is particularly critical for maintaining the peak brightness temperature values of sub-pixel fire hotspots, which would otherwise be attenuated by bilinear or cubic interpolation.

### 2.2. Validation Data: FRP and EFFIS

FRP data for validation are stored in a custom geodatabase, updated daily since 2022. The dataset includes active fire products from MODIS (C6.1), VIIRS (C2), and SLSTR sensors, carried aboard the Terra/Aqua, Suomi-NPP, NOAA-20/21, and Sentinel-3 platforms, respectively. These products are automatically downloaded to provide a radiometric reference for MTG-FCI detections. Because of their polar orbits, these sensors provide intermittent confirmations only during specific daily overpasses. We also used fire perimeters from the European Forest Fire Information System (EFFIS) as a spatial reference. These polygons represent the final burned areas and are used to confirm the location of the detected pixels. Although EFFIS perimeters are often refined after the event using high-resolution imagery, they provide a necessary spatial reference to confirm the hotspots identified by the algorithms.

### 2.3. Fire Detection Methodologies

Active fires are detected using two complementary unsupervised approaches applied to MTG-FCI data. The first is a classical threshold-based method, in which spectral thresholds are applied to identify thermal peaks. The second is an experimental approach based on a Lightweight U-Net autoencoder, trained to reconstruct the normal background; fires are then detected as statistical anomalies in the reconstruction error. This choice allows testing two fundamentally different unsupervised strategies with MTG-FCI observations, and the goal is to assess their feasibility rather than to determine which method is superior.

### 2.4. Threshold Methodology

Similar to earlier satellite-based fire detection algorithms, our method utilizes the varying responses of middle-infrared and long-wave-infrared bands to scenes with hot subpixel targets [26]. The threshold methodology is based on two values, a threshold temperature and a difference value applied to the entire domain used, which is the Italian Peninsula in our case. The threshold value is applied to the MWIR band, which results sensitive to high-temperature sub-pixel sources. The TIR measures the actual surface temperature. It is less affected by solar reflection and provides a stable baseline for the Earth’s ambient thermal emission. The formula used for this first approach to the threshold methodology is

(1) A=(MWIR>GT)∧(MWIR−TIR>DGT)

where GT represents the global threshold used in the domain. This threshold acts as a trigger to identify “hot” pixels while filtering out standard ground temperatures. According to Wien’s Displacement Law, as a fire’s temperature increases (600–1000 K), its peak emission shifts toward this shorter wavelength. Therefore, a fire increases the radiance at MWIR much more significantly than at TIR, a high value of the difference DGT confirms a combustion source rather than uniform solar heating of the ground. The value of GT used was chosen between the maximum between 322 K and the 99.99th percentile of the temperature distribution. While 322 K proved effective during the month of June, it created many false positive contiguous pixels in warmer areas of Southern Italy during the month of August. The introduction of a max value between 322 K and that 99.99th percentile allowed us to avoid those false positives. The value of DGT was set to 12 K.

### 2.5. Bottleneck Constrained Light U-Net Autoencoder

Unlike the thresholding method, the CNN model requires an additional normalization step to process all channels consistently. All bands are normalized to the range [0, 1] based on their respective minimum and maximum values:

(2) $$x_{b}^{\prime }=\frac{x_{b}-x_{b\min}}{x_{b\max}-x_{b\min}+\varepsilon }$$

where $x_{b}^{\prime }$ is the normalized value, $x_{b}$ is the row value, and $x_{b\min}$ and $x_{b\max}$ are the minimum and maximum reference values established for band *b*, respectively. This step is crucial because the IR bands are in brightness temperature while the NIR bands are in reflectance, and normalization ensures that the model can process all channels consistently.

The Bottleneck-constrained Light U-Net (BLU-Net) is a Convolutional Neural Network (CNN) model, specifically a modified U-Net [27] architecture, for anomaly detection [28]. The model is trained on fire-free images to learn the reconstruction of typical landscape structures. Fire events generate a high reconstruction error compared to the learned background, allowing for the spatial localization of the anomaly [29].

The method assumes that complex images can be represented by a lower-dimensional manifold [30]. By constraining the bottleneck to a limited number of channels, the model is forced to prioritize invariant terrain features while discarding non-essential variations for background reconstruction. To evaluate the approach’s feasibility, the model was trained using a subset of spectral bands in this first experimental phase.

#### 2.5.1. Architecture

The proposed architecture is designed to balance structural compression with reconstructive precision. While standard deep models risk overfitting to anomalies, this lightweight configuration acts as a selective filter, focusing on the essential structures of the image while leaving unusual features unreconstructed. Instead of standard multi-channel detection, the autoencoder captures natural variations in the landscape, such as clouds, hot surfaces, or ground reflections. The autoencoder characterizes the nominal background variance; consequently, transient thermal anomalies result in high reconstruction residuals.

**Figure 1:**
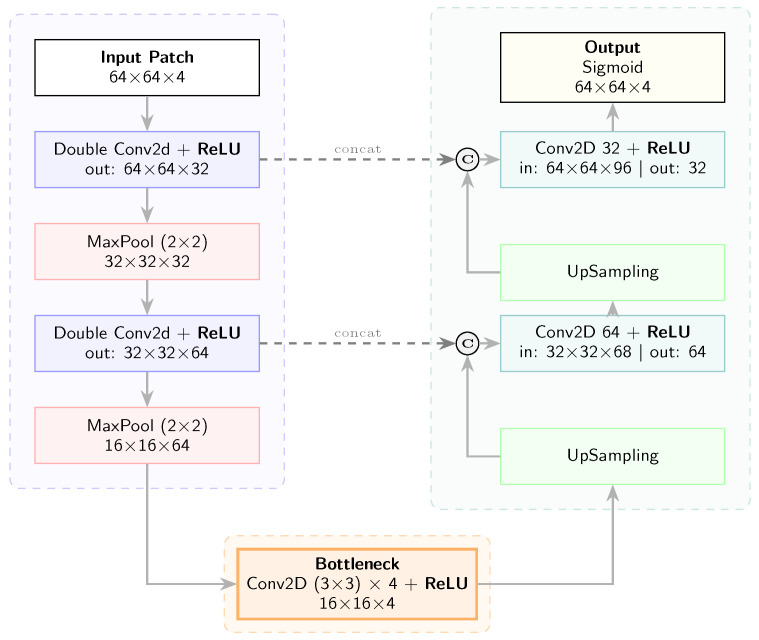
Block diagram of the BLU-Net model. The architecture shows a 4-channel bottleneck connecting the encoder and decoder. Dashed lines represent skip connections with concatenation (©) to recover spatial details, with a Sigmoid activation producing the final 64×64×4 output.

To preserve edge definition and restore spatial sharpness, the decoder recovers details directly from the encoder using skip connections [31]. The encoder–bottleneck–decoder architecture with skip connections is shown in Figure 1. Within the encoder, each block of double convolutions is followed by a 2×2 Max Pooling layer. This operation progressively reduces the spatial dimensionality of the feature maps from the initial 64×64×32 representation to 16×16×64 before reaching the 16×16×4 constrained bottleneck.

This compression forces the network to learn the most significant features of the fire-free background before the decoder reconstructs the original patch size through upsampling and skip connections. Driven by Max Pooling, this architectural compression drastically reduces the number of trainable parameters to only ∼136 k, allowing fast training on 64×64 patches with 4 spectral bands. This lightweight layout enables the model to expand the receptive field of subsequent layers without increasing the computational load, making the entire system highly suitable for near-real-time (NRT) processing.

To ensure geographic consistency while strictly avoiding data leakage, the training dataset was extracted from the exact same geographic domain (the Italian territory) but during a temporal window distinct from the June–August validation period. Specifically, fire-free imagery was selected from the months of May and late September 2025. This choice allows the model to characterize the nominal surface background and transitional seasonal variations without being exposed to peak summer wildfire signatures.

Furthermore, the training dataset was carefully balanced to include both daytime and nighttime acquisitions to capture both diurnal and nocturnal thermal dynamics. Notably, cloudy scenes were intentionally included in the training loop. This strategy forces the autoencoder to reconstruct transient clouds as part of the ‘normal’ atmospheric background state, ensuring that they yield minimal reconstruction error during inference and preventing them from being misclassified as outliers. Using only on fire-free samples, selected across different hours of the day and varying cloud conditions, the model becomes highly specialized in reconstructing the nominal state of the terrain; consequently, any anomaly or fire-related feature in new data will result in higher reconstruction residual that the bottleneck cannot encode, highlighting the deviation from the learned background.

Through an empirical ablation study on d∈{1,2,4,6,8,16}, the architectural impact of the bottleneck dimension was quantified. To assess how the latent space constraint translates into downstream detection quality, the model’s anomaly maps were binarized using the logic detailed in Section 2.5.2 over a validation subset synchronized with the Fire Radiative Power (FRP) data. Detections were classified into True Positives (TP), False Positives (FP), and False Negatives (FN) based on spatial intersection with the buffered FRP clusters, while True Negatives (TN) were computed across the remaining image-wide nominal background pixels. Within this validation framework, the classification performance is evaluated using Sensitivity, as defined in Equation (3), the False Positive Rate (FPR), expressed in Equation (4), and the balanced F1-Score, formulated in Equation (5):


(3) $$\mathrm{Sensitivity}\;\left(\mathrm{TPR}\right)=\frac{TP}{TP+FN}$$

(4) $$\mathrm{FPR}=\frac{FP}{FP+TN}$$

(5) $$F1\text{-}\mathrm{Score}=\frac{2\cdot TP}{2\cdot TP+FP+FN}$$

The results of this sensitivity analysis are summarized in Table 2. Restricting the bottleneck to d=1 or d=2 causes structural underfitting; the network fails to resolve standard environmental features, yielding high reconstruction residuals ($7.9\times 10^{-3}$ for d=1) and inflating the False Positive Rate (FPR) up to 14.82%.

Conversely, expanding the bottleneck to d=6, d=8, or d=16 minimizes the training metrics but triggers an anomaly leakage effect. The excessive degrees of freedom allow the network to encode and reconstruct the high-intensity thermal anomalies. Consequently, the residual error on active fires drops during inference, causing the sensitivity to collapse down to 9.0–27.0% for d=16. The configuration d=4 represents the mathematical optimum for unsupervised anomaly detection, maximizing the F1-Score (78.5–83.2%) by suppressing background noise without reconstructing the wildfire signatures.

This bottleneck compression forces the network to extract the most stable spatial and spectral features of the landscape. This dimensionality reduction prevents pixel-level memorization and ensures that the decoder reconstructs only the permanent background, treating transient elements like fires as reconstruction noise.

To support this compression and prevent the encoder from blurring sharp variations within the image, simple Rectified Linear Unit (ReLU) activation functions are used, to map complex relationships between pixels.

The training process employs the Huber loss [32] with δ=0.05 as a robustness–precision trade-off. This approach follows the principle of robust statistics, where the loss function is designed to be less sensitive to outliers [33,34] which would otherwise skew the background manifold reconstruction [35].

**Figure 2:**
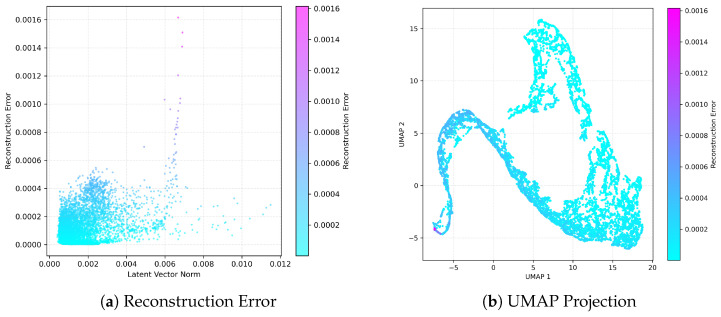
(**a**) Reconstruction error as a function of the latent vector norm, showing a clear separation of anomalies (high error) from the normal terrain background. (**b**) UMAP projection of the latent manifold, where fire-related features (purple) form a distinct, localized cluster, confirming the model’s ability to isolate outliers from the diverse patterns of the natural landscape.

Figure 2a illustrates that anomalies result in significantly higher reconstruction errors compared to the training distribution. This happens because the narrow bottleneck acts as a sort of selective filter: it forces the network to focus on familiar landscape patterns, effectively ignoring the fire-related information it cannot compress. The UMAP projection (Figure 2b) shows fire features (purple dots) forming a compact, isolated cluster rather than dispersing randomly. This indicates that the model maps these anomalies to a distinct region of the manifold, making them easy to separate from the background using a simple reconstruction-error threshold.

This structural behavior provides the theoretical rationale for the BLU-Net architecture design. Standard U-Net models possess excessive parameter capacity, which behaves similarly to the oversized latent spaces (d≥8). In an unsupervised anomaly detection framework, an over-parameterized network acts as an unconstrained identity mapping, reconstructing both the nominal background and transient anomalies with minimal residual differentiation. To qualitatively contextualize this choice against common unsupervised baselines, anomaly segmentation typically faces a strict trade-off between standard Convolutional Autoencoders (CAEs) without skip connections and standard deep U-Nets. While a classic CAE effectively prevents anomaly leakage due to the absence of high-frequency shortcuts, its structural layout causes severe spatial oversmoothing, failing to reconstruct complex nominal background elements (e.g., sharp coastlines or dynamic cloud boundaries) and thus inflating false alarms. Conversely, a standard U-Net preserves background sharpness but triggers complete anomaly leakage. By reducing the parameter space to approximately 136 k parameters and constraining the bottleneck channels, the proposed BLU-Net structurally reconciles this trade-off. This architecture constraint ensures that the latent manifold encodes only the low-frequency nominal background patterns, while the lightweight skip connections preserve environmental sharpness, forcing active fires to emerge as high-residual reconstruction outliers during inference. Furthermore, scaling the architecture to a standard deep U-Net would not only exacerbate this anomaly leakage due to parameter inflation, but would also violate the operational constraints of near-real-time geostationary processing. While a standard heavy U-Net increases the computational latency and hardware requirements, the optimized 136 k parameter layout of BLU-Net ensures low-latency inference, executing 500 times faster than the MTG-FCI acquisition rate without sacrificing detection performance.

#### 2.5.2. Detection Strategy

We explore the feasibility of unsupervised wildfire detection using the MWIR thermal infrared band [36] from MTG-FCI data. Detection is framed as a dual-scale contextual anomaly problem. The input images are processed using a moving window strategy $W_{x,y}$ of size 64×64 pixels with 50% overlap (stride =32), in order to preserve spatial continuity. Each patch is reconstructed by the model and the final image $\hat{I}$ is obtained by uniformly averaging the overlapping reconstructions [37], according to

(6) $$\hat{I}\left(x,y\right)=\frac{\sum_{k}{\hat{P}}_{k}\left(x,y\right)}{\sum_{k}w_{k}\left(x,y\right)},$$

where ${\hat{P}}_{k}$ represents the reconstruction of the *k*-th patch and $w_{k}\left(x,y\right)$ denotes the number of contributions at pixel (x,y). This procedure reduces edge artifacts introduced by inference and ensures spatial consistency in the reconstructed image.

**Figure 3:**
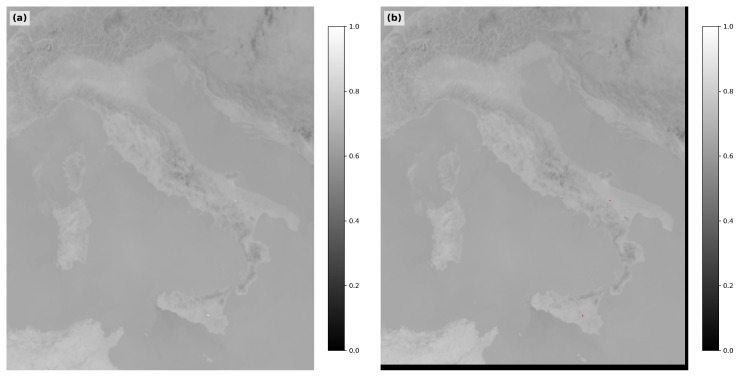
Qualitative detection results. (**a**) Original normalized MWIR band. (**b**) Reconstructed background using the BLU-Net with the final fire mask overlaid in red. The black margins in the reconstructed image (**b**) are due to the 64×64 pixel windowing process, which excludes border pixels not fitting the fixed stride. Contains modified EUMETSAT data.

The black margins visible at the absolute edges of the final reconstruction (e.g., Figure 3b) represent a deterministic artifact of this grid layout. When the total dimensions of the input scene are not perfectly divisible by the 32-pixel stride, the remaining border pixels (at most 31 pixels on the right and bottom boundaries) cannot accommodate a full (64×64) window and are excluded from the inference grid. To prevent any data degradation, the geographic crop of the scene was intentionally defined so that these unmapped margins fall entirely outside the Italian territory, primarily over marine areas. Consequently, there is zero data loss within the study area, and the reconstruction error remains mathematically uniform up to the edge of the active processing grid.

Based on this reconstruction, two complementary indicators are considered: the reconstruction error,

(7) $$E\left(x,y\right)=\left|I\left(x,y\right)-\hat{I}\left(x,y\right)\right|,$$

and the radiometric intensity of the MWIR band. To account for the spatial heterogeneity of the background (e.g., vegetation, urban areas, and naturally hot surfaces), both quantities are standardized within the local 64×64 pixel window. Specifically, we compute local z-scores for each patch: $z_{e}$ for the reconstruction error and $z_{h}$ for the thermal intensity of the MWIR band. These standardized indicators quantify how much each pixel deviates from its local neighborhood in terms of reconstruction anomaly and thermal excess.

The two contributions are then combined into a local score to capture joint positive deviations:

(8) $$S_{\mathrm{local}}\left(x,y\right)=\alpha \max\left(0,z_{e}\left(x,y\right)\right)+\left(1-\alpha \right)\max\left(0,z_{h}\left(x,y\right)\right),$$

where α is a weighting coefficient set to 0.7. This value was empirically selected during the tuning phase to give dominant weight to the autoencoder’s reconstruction. Preliminary tests showed that shifting this balance toward the local or global background components significantly increased false positives. Specifically, minor variations triggered false alarms near cloud boundaries during early morning hours and over industrial or highly anthropized zones during peak diurnal heating. The 0.7 ratio was found to be the optimal empirical trade-off to suppress these localized noise sources. While $S_{\mathrm{local}}$ provides a continuous measure of joint anomaly strength, for this feasibility study we evaluate threshold-based detection by applying percentile-based constraints independently to the two standardized indicators within the region of interest.

The binarization thresholds for the global ($p_{g}$) and local ($p_{e},p_{h}$) percentile constraints were calibrated through a grid-search analysis, testing $p_{g}\in \left\{99.9,99.95,99.99\right\}$ and $p_{e},p_{h}\in \left\{91,93,95,97,99\right\}$. The results indicated that maximizing $p_{g}$ to 99.99 neutralizes the spatial regularizer, forcing a uniform F1-score of 0.527 across all local configurations. To preserve the functional contribution of the local contextual score, $p_{g}=99.95$ was selected. Within this configuration, setting $p_{e},p_{h}=95$ positions the framework at the center of a stable performance plateau yielding an F1-score of 0.522, ensuring robustness against minor background fluctuations.

To reduce overdetection in globally noisy or thermally active scenes, the combined score is standardized at the scene level:

(9) $$z_{g}\left(x,y\right)=\frac{S_{\mathrm{local}}\left(x,y\right)-\mu_{\mathrm{scene}}}{\sigma_{\mathrm{scene}}},$$

where $\mu_{\mathrm{scene}}$ and $\sigma_{\mathrm{scene}}$ denote the mean and standard deviation of $S_{\mathrm{local}}$ computed over the region of interest.

The final fire mask is defined through a conjunctive, triple percentile-based constraint applied to both local indicators and the global scene-level score:

(10) $$F\left(x,y\right)=1\left(z_{e}\left(x,y\right)\geq P_{p_{e}}\left(z_{e}\right)\;\wedge \;z_{h}\left(x,y\right)\geq P_{p_{h}}\left(z_{h}\right)\;\wedge \;z_{g}\left(x,y\right)\geq P_{p_{g}}\left(z_{g}\right)\right),$$

where $P_{p_{e}}\left(\cdot \right)$ and $P_{p_{h}}\left(\cdot \right)$ denote the $p_{e}$-th and $p_{h}$-th percentiles of the local distributions, and $P_{p_{g}}\left(\cdot \right)$ denotes the $p_{g}$-th percentile of the scene-level distribution. This conjunctive strategy prioritizes precision by retaining only pixels that consistently fall within the extreme tails of all three indicators. In the adopted configuration, $p_{e}=95$, $p_{h}=95$, and $p_{g}=99.95$.

Overall, this procedure combines the selective reconstruction capabilities of the BLU-Net with a local statistical analysis. Although the model currently uses only a subset of spectral bands, these preliminary results suggest that MTG-FCI observations contain sufficient information to support unsupervised detection of wildfire hot spots. As shown in Figure 3, the system reconstructs both the terrestrial background and the nominal atmospheric state, including cloud patterns. By learning the statistical distribution of the combined radiance, the model removes the need for explicit cloud masking. In this framework, transient clouds are reconstructed as part of the ‘normal’ state, resulting in minimal reconstruction error, whereas stationary thermal anomalies, such as wildfires, produce a localized spectral divergence that triggers the detection mechanism.

## 3. Results and Discussion

The results section is structured into two complementary validation phases to evaluate the performance of the unsupervised architectures. First, a localized, qualitative assessment is presented through selected multi-temporal case studies to observe the algorithms’ spatial behavior and boundary stability under different environmental conditions. Second, to prevent local sampling bias and provide an objective system evaluation, a rigorous statistical aggregation is conducted. This quantitative phase macroscopically assesses the algorithms’ performance across the entire 11-day dataset using standardized metrics, including precision, recall, $F_{1}$-score, and temporal latency distributions.

### 3.1. Validation Framework and Ground-Truth Limitations

The validation of the results faces a fundamental challenge: the absence of an absolute “ground truth”. The reference datasets commonly used, although authoritative, possess structural limitations documented in the literature that make a direct comparison difficult:

- Active Fire products (MODIS, VIIRS, SLSTR): These sensors carry several false positives (errors of commission). As demonstrated by Filipponi and Mercatini [38], many of these alerts are not real fires, but thermal anomaly hot spots misclassified as fire pixels caused by industrial sites or by solar reflections on roofs like solar plants.
- The EFFIS minimum threshold: Although it is a manually validated database, EFFIS focuses almost exclusively on large fires (usually over 30 hectares). This means that small outbreaks, which are the most difficult and important to monitor immediately, often remain invisible in the reference maps [39].
- The time factor (LEO vs. GEO): Polar satellites pass overhead only a few times a day. This creates a "time gap" compared to the continuous monitoring of GEO satellites: if the fire ignites or dies out between passes, the polar satellite risks missing the peak intensity or not recording the event at all [40].

To address the lack of a continuous validation dataset, we adopted a multi-source strategy [41]. Each hotspot detected by our unsupervised algorithms was systematically cross-referenced with active fire detections from MODIS/VIIRS and the EFFIS database. In cases where our models identified fires not reported by these conventional systems, we performed a targeted pre- and post-event analysis using Sentinel-2 MultiSpectral Instrument (MSI) imagery. The 20 m spatial resolution of MSI allowed us to confirm the presence of fire scars exactly at the coordinates detected by MTG-FCI, proving the sensitivity of our approach to small outbreaks that often fall below the minimum mapping thresholds of EFFIS. However, a notable technical constraint is the lack of a perfect temporal coincidence between satellite passages. While MTG-FCI detects thermal anomalies in NRT, the Sentinel-2 overpass typically occurs within a window ranging from 2 to 3 days at mid-latitudes. This time gap means we cannot always guarantee that a specific hotspot is the exact same ignition event that visible scar. Nevertheless, the precise spatial match between our detections and the subsequent appearance of a burn scar provides a level of confidence that standard databases cannot offer, confirming the operational potential of unsupervised monitoring.

To formalize this validation within a strict statistical framework, a quantitative assessment was conducted by benchmark-referencing our detections exclusively against the operational active fire products (FRP). Consequently, the resulting classification errors were systematically categorized into standard metrics, including precision, recall, False Alarm Rate (FAR), and $F_{1}$-score, as detailed in Section 3.3.

It must be emphasized that using FRP products as the sole numerical ground truth represents a highly conservative evaluation approach. As demonstrated by our complementary dNBR spatial analysis, several detections classified as False Positives under this strict regime correspond to localized burn scars, confirming the occurrence of physical fire events omitted by the reference FRP products. Therefore, the quantitative metrics presented hereafter should be interpreted as a conservative lower bound of the actual algorithm performance.

### 3.2. Spatial Consistency and Qualitative Case Studies

To ensure a consistent comparison, both methods were evaluated on a daily basis. BLU-Net produces a detection mask for each MTG-FCI frame, while the Thresholding method generates a single mask from the daily image stack. The individual BLU-Net masks were therefore merged into a daily composite mask, allowing both methods to represent the total area affected by fire activity over the same 24 h period.

Within this framework, a fire event is operationally defined as a spatially contiguous cluster of detected fire pixels extracted from these daily composite masks.

Although covering 11 calendar days, the geostationary sampling frequency (144 acquisitions per day) provides a total of 1584 multispectral scenes. This dataset captures diurnal thermal transitions and cloud dynamics over the study area. Furthermore, this temporal window encompasses 349 independent ground-truth fire events validated against polar overpasses, providing a representative sample size for this feasibility study.

The spatial evaluation focuses on a dataset of 11 selected days tracked during the 2025 Italian summer fire season. While the August dates were chosen to coincide with major wildfire events on Mount Vesuvius and surrounding regions, all other dates in the series were strategically selected because they present a wide variety of concurrent fire events across different geographic areas, providing a robust dataset for evaluation. Across these 11 days, the daily composite masks from both the Thresholding method and the BLU-Net architecture were systematically extracted to observe their local spatial boundaries against the Sentinel-2 dNBR imagery, before submitting the entire multi-temporal series to the rigorous cross-validation framework detailed in Section 3.3.

**Figure 4:**
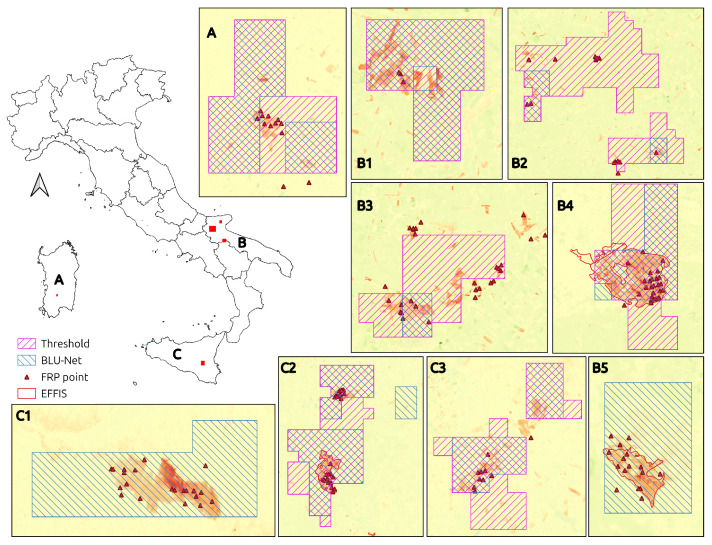
Spatial consistency for the 21 July 2025 event shows localized events in regions A (Sardinia), B (Apulia), and C (Sicily). Panels (**A**,**B1**–**B5**,**C1**–**C3**) demonstrate the agreement between BLU-Net (blue grid) and Thresholding (magenta grid) masks compared to validation data (FRP points/red triangles, EFFIS perimeters/red outlines) over Sentinel-2 dNBR background, using modified Copernicus data.

**Figure 5:**
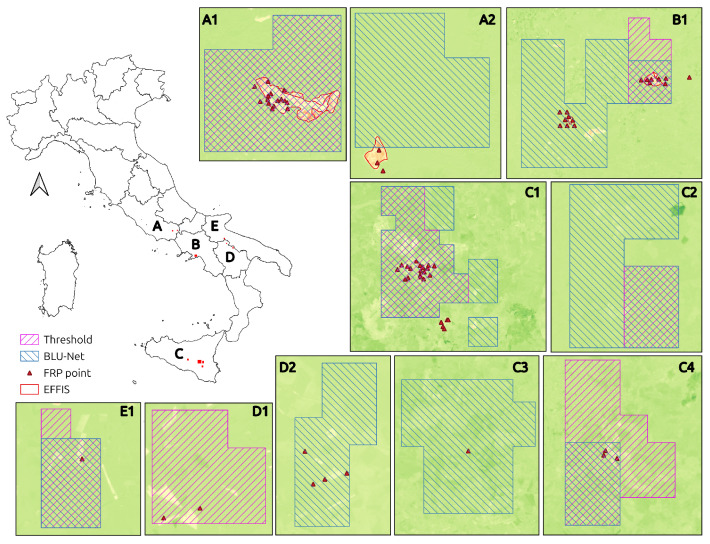
Spatial consistency for the 12 August 2025 event across regions A (Lazio), B (Campania), C (Sicily), D (Basilicata), and E (Apulia). Panels (**A1**,**A2**,**B1**,**C1**–**C4**,**D1**,**D2**,**E1**) compare BLU-Net (blue grid) and Thresholding (magenta grid) masks against validation data (FRP triangles, EFFIS outlines) over Sentinel-2 dNBR background. Contains modified Copernicus data.

Figure 4 and Figure 5 show some representative fire events observed on 21 July and 12 August 2025, illustrating BLU-Net masks (blue), Thresholding method masks (magenta), FRP points (red triangles), and EFFIS perimeters where available (red outlines). The background displays differenced Normalized Burn Ratio (dNBR false color) derived from pre- and post-event Sentinel-2 imagery.

In Figure 4, panels A, B1, B2 and B3 show several events where the BLU-Net and Thresholding method masks spatially overlap. These detections coincide with small clusters of FRP points. The background dNBR layer clearly shows the burned areas associated with these locations (in red). In panel B4, a large fire event is shown. The burned area is clearly visible in the dNBR background and aligns with both the EFFIS perimeter and clusters of FRP points. This event is detected by both BLU-Net and the Thresholding method. Panel B5 shows a fire event clearly identified by the dNBR background, featuring a cluster of FRP points and an EFFIS perimeter. Both BLU-Net and the Threshold method correctly detect this event. Panels C2 and C3 show multiple detections, in C2 two fire events are correctly identified by both methods, confirmed by Effis polygon and FRP clusters. In the upper-right section a small grassland or agricultural fire, likely transient, is detected only by BLU-Net. In panel C1, a fire confirmed by dNBR and FRP points, but absent from EFFIS, is detected only by BLU-Net.

In Figure 5, panels A1, A2, and B1 show several events identified by both EFFIS and FRP points. In panel A1, BLU-Net and the threshold method overlap perfectly. In panel A2, the fire is detected only by BLU-Net. In panel B1, both methods identify the larger fire in the top right, while only BLU-Net detects the smaller one on the left. In panel C1, both BLU-Net and the threshold method identify a larger event, confirmed by the yellow-orange dNBR signal and FRP points. In the bottom right, a much smaller fire is detected only by BLU-Net. In panel C2, both BLU-Net and the threshold method identify an event characterized by a barely perceptible dNBR variation, in the absence of both FRP points and EFFIS polygons, most likely due to a low-intensity fire of very short duration. In panel C4, both methods identify a fire characterized by only 3 FRP points and a slight variation in dNBR. In panels C3 and D2, only the BLU-Net method identifies two very weak events, characterized by the presence of a few FRP points and barely visible against the dNBR background. In panel D1, conversely, only the threshold method identifies a fire, likely related to a agricultural fire, detectable in both the dNBR and through two FRP points. In panel E1, both methods show an almost perfect overlap on a small grassland event, detectable through the dNBR and the presence of a single FRP point.

#### 3.2.1. Validation of MTG-FCI Detections Using Sentinel-2: The Foggia Fire Event

The Foggia fire, observed on 7 June 2025, illustrates the detection of small-scale or transient events, which can often be mistaken for instrumental noise or false positives. In this instance, the event took place in the Apulia region (Southern Italy), specifically in the province of Foggia. It involved a cluster of multiple detections, with the largest burned area polygon covering approximately 50 hectares, surrounded by 3 other smaller fire scars. Despite the overall extent, the event was not recorded by EFFIS polygons, likely due to its exclusively agricultural nature or its proximity to an urban area, factors that can sometimes exclude events from wildfire-specific databases.

**Figure 6:**
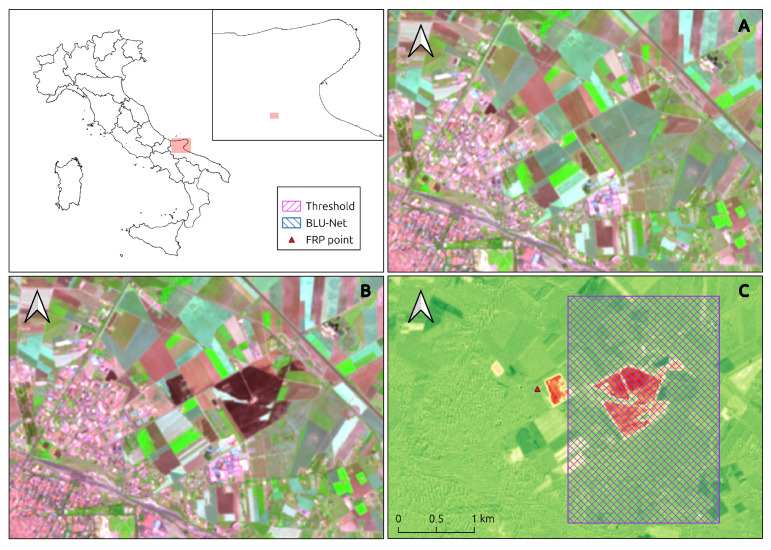
Validation of a transient fire event in Foggia (7 June 2025). (**A**) Pre-fire and (**B**) post-fire Sentinel-2 false-color composites (B12-B8A-B04). (**C**) Spatial comparison between the Sentinel-2 dNBR (background), the BLU-Net detections (blue grid), the Thresholding method (magenta grid), and the FRP point (red triangle). This contains modified Copernicus Sentinel data.

The temporal dynamics are particularly revealing: while the MODIS Aqua FRP product (red triangle in Figure 6C) identified a hotspot at 13:10 UTC, the fire was identified in a single MTG-FCI image at 13:40 UTC, suggesting a short duration event. To validate these detections, high-resolution Sentinel-2 imagery was used, with the pre-fire reference acquired on 7 June 2025 (between 10:00 and 12:00 UTC) and the post-fire image on 10 June 2025 (between 09:50 and 12:00 UTC). The False Color (Bands 12, 8A and 4), in Figure 6A,B, clearly reveal the burn scars, showing a sharp contrast between the healthy vegetation and the charred areas. The spectral evidence from the dNBR analysis (Figure 6C), confirms that the anomalies identified by BLU-Net and the threshold method at 13:40 UTC were not false alarms, but a series of real fire events, demonstrating the crucial role of MTG’s high temporal cadence in resolving transient phenomena that might otherwise remain unclassified.

#### 3.2.2. 5–11 August 2025: The Vesuvius Fire Event

Another noteworthy event was the large fire that affected the southeastern slope of Vesuvius in August. The fire was first detected by BLU-Net and Thresholding method at 14:20 UTC on 5 August 2025, and showed intermittent activity until 19:10 UTC. In contrast, the first available Fire Radiative Power data were recorded between 19:45 and 20:24 UTC.

**Figure 7:**
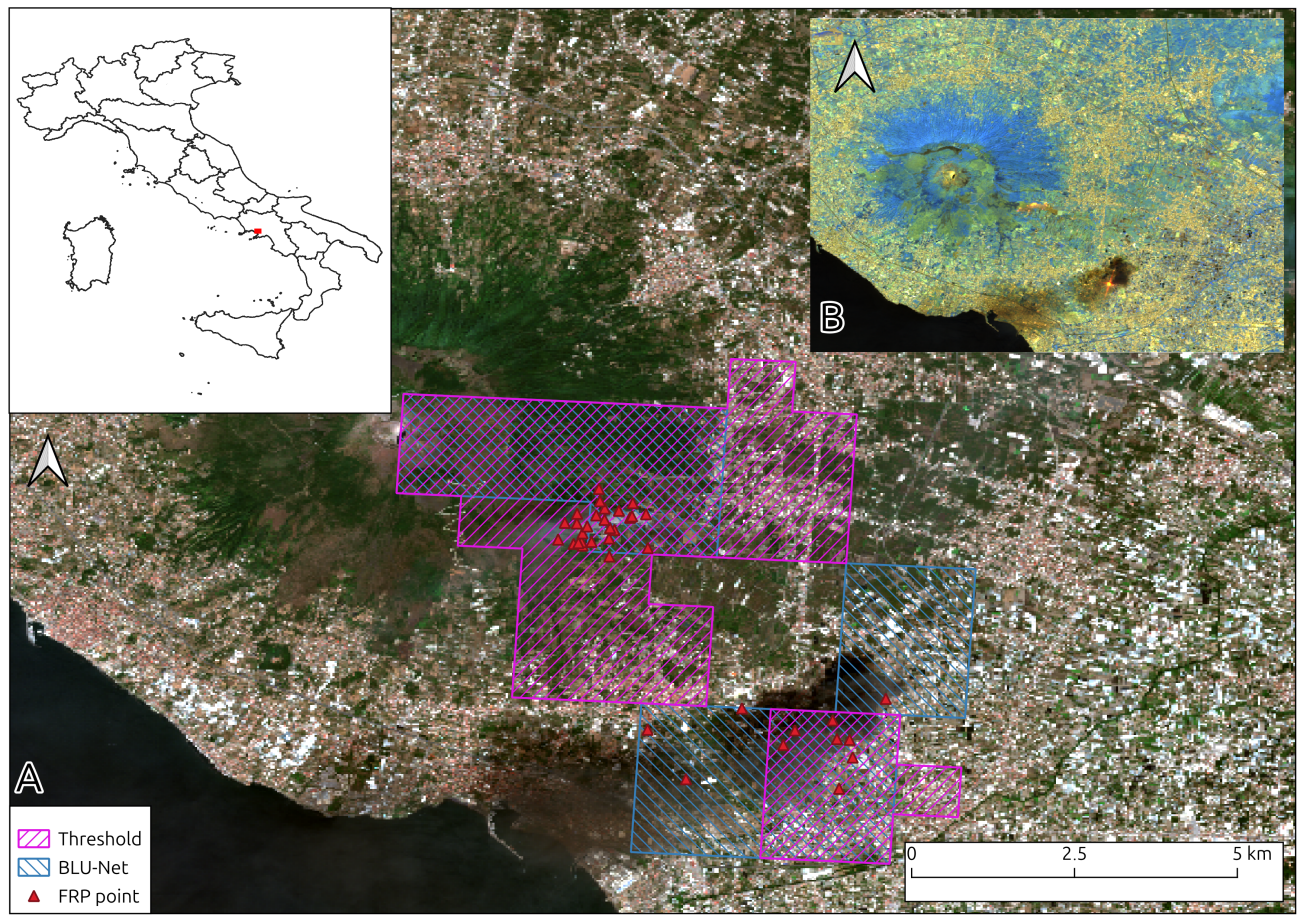
Vesuvius fire event, focusing on the second day of activity (6 August 2025). (**A**) Sentinel-2 True Color composites (B04-B03-B02) in background, the BLU-Net detections (blue grid), the Thresholding method (magenta grid), and the FRP point (red triangle). (**B**) Sentinel-2 Swir composites (B12-B11-B8A). This contains modified Copernicus Sentinel data.

The event’s progression was further validated using a Sentinel-2 acquisition on 6 August, which confirmed the spatial extent of the burn scar and the persistence of the front. The algorithms successfully isolated the wildfire’s structural anomaly even in the presence of a secondary industrial fire located in the proximity. Despite its limited size, this second event was correctly identified and isolated by both BLU-Net and the Thresholding method (Figure 7).

On 7 August, neither BLU-Net nor the Thresholding method recorded any significant detections. The EFFIS database includes the fire polygon only two days after the event began. However, only two isolated FRP points were detected in the southernmost part of the scar. This suggests that the EFFIS polygon for 7 August likely represents a post-event update rather than indicating an active fire front.

On 8 August, a significant reactivation of the fire was observed. Both BLU-Net and the Thresholding method detected the event in 31 MTG-FCI images, spanning the time window from 15:40 UTC to 23:50 UTC. This activity corresponds with a high density of FRP points, totaling 32, recorded along the southeastern slope, all of which fell within the detection masks of both methods. The recorded FRP data were exclusively from nighttime passes. In relation to the EFFIS database, the stored polygon does not facilitate a daily estimation of the fire front’s progression, as it appears to have been refined after the event, already encompassing the wildfire’s final perimeter.

**Figure 8:**
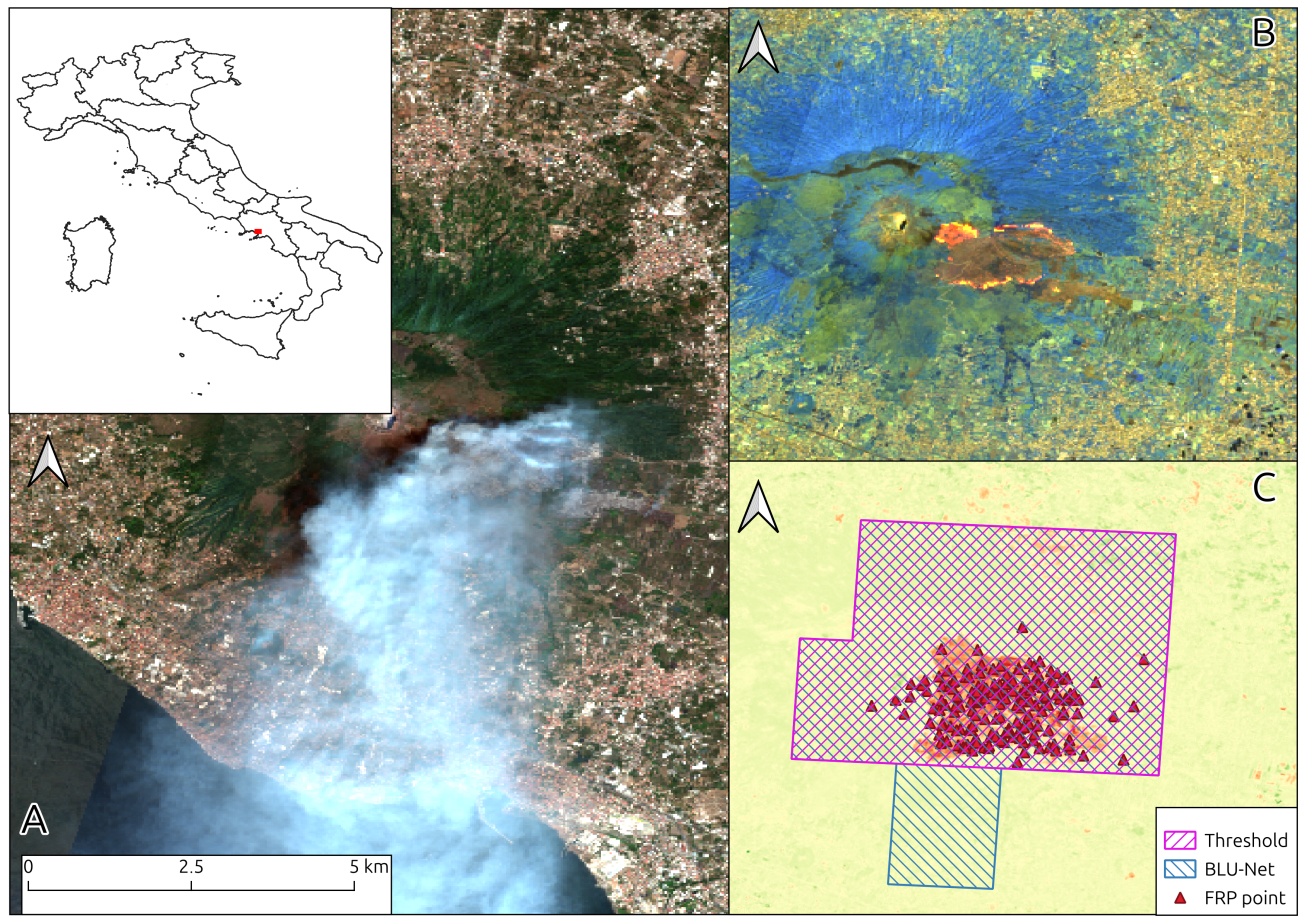
Vesuvius fire event, focusing on the peak activity day (9 August 2025). (**A**) Sentinel-2 True Color composite (B04-B03-B02) highlighting the smoke plume. (**B**) Sentinel-2 SWIR composite (B12-B11-B8A) revealing the active fire fronts through the smoke. (**C**) Validation map with SWIR background showing the overlap between BLU-Net detections (blue grid), the Thresholding method (magenta grid), and the FRP points (red triangles). This contains modified Copernicus Sentinel data.

On 9 August 2025, the fire showed its maximum intensity and persistence. As shown in the Sentinel-2 True Color composite (Figure 8A), a dense smoke plume covers the area, which is instead penetrated by the SWIR imagery to reveal the active fire fronts (Figure 8B). The BLU-Net model highlighted a detection in every single MTG-FCI image acquired during the day, for a total of 144 images. This temporal continuity is extremely robustly confirmed by the radiometric data: on the southeastern slope of Vesuvius, 168 FRP points were recorded over the course of 24 h. These points consistently fell within the detection masks generated by both BLU-Net and the Thresholding method (Figure 8C).

On 10 August 2025, the fire activity showed a significant decrease. The event was detected by BLU-Net in 37 MTG-FCI images, specifically from 00:10 UTC to 05:30 UTC, with some discontinuities, and again from 20:30 UTC to 23:50 UTC. A total of 62 FRP points were recorded on the volcano’s slopes, primarily distributed along the edges of the burned area. In this phase, the Thresholding method mask appears more compact, while the BLU-Net detection tends to expand toward the south. In both cases, almost all recorded FRP points fell within the masks generated by the two methods.

On 11 August 2025, the situation remained similar to the previous day. BLU-Net detected the fire in 65 MTG-FCI images, specifically from 00:00 UTC to 14:50 UTC, with some discontinuities. A total of 52 FRP points were recorded on the volcano’s slopes. In this case, the detection masks produced by both BLU-Net and the Thresholding method showed a spatial distribution similar to that of 10 August, with almost all FRP points falling within their respective perimeters.

#### 3.2.3. Detection of Industrial Fire: Catania Fire Event

In the Catania case study, observed on 9 August 2025, an industrial fire was detected by BLU-Net in four consecutive MTG-FCI images (08:10–08:40 UTC), while no detections were triggered by the Thresholding method. The event is confirmed by a Sentinel-2 overpass at approximately 10:00 UTC.

**Figure 9:**
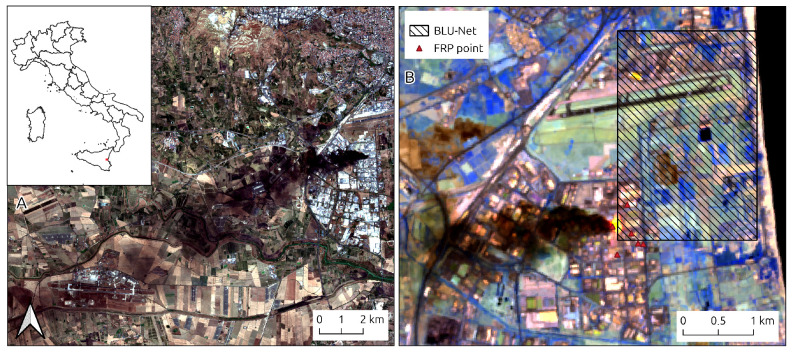
Industrial fire event in Catania; 9 August 2025: (**A**) Sentinel-2 True Color composite (B04-B03-B02) showing the smoke plume over the industrial area. (**B**) Sentinel-2 SWIR composite (B12-B11-B8A) where the active fire front (bright pixels) is clearly visible, with overlay: BLU-Net detection (hatched grid) and FRP points (red triangles). This contains modified Copernicus Sentinel data.

The True Color composite (Figure 9A) shows a dense black smoke plume and the SWIR composite (Figure 9B) reveals the active fire front. Despite the sub-pixel dimensions of the fire (estimated at around $1000\;m^{2}$), it was correctly identified by the model. Six FRP points, strongly correlated with a BLU-Net mask, were recorded only later in the day, between 11:08 and 20:21 UTC. This demonstrates the model’s ability to detect localized thermal anomalies in complex backgrounds several hours before they are recorded by polar-orbiting sensors.

### 3.3. Quantitative Performance Assessment

To quantify detection performance, the algorithms polygons were validated against FRP. To exclude isolated thermal anomalies caused by solar reflections or anthropogenic surfaces, only FRP clusters containing at least two points were considered valid ground-truth events. Furthermore, a 1 km buffer was applied around the FRP coordinates to compensate for spatial resolution mismatches and parallax positioning errors.

The evaluation metrics were defined based on the following spatial intersection criteria: unlike the pixel-level sensitivity analysis detailed in Section 2.5.1, where True Negatives (TN) are computed across the continuous image background to derive the False Positive Rate, the macroscale performance assessment is formulated strictly as an object-based validation against discrete reference points. Within this object-centric baseline, True Negatives are inherently undefined, as it is mathematically impractical to quantify non-events or non-existent fire clusters that the algorithms correctly omitted. Consequently, the performance evaluation relies exclusively on classification metrics that do not require TN formulations, namely precision, recall, and the cumulative $F_{1}$-score, determined by the following object-level intersection rules:

- True Positives (TP): Algorithm detection polygons intersecting a buffered FRP cluster.
- False Positives (FP): Algorithm detection polygons failing to intersect any buffered FRP cluster.
- False Negatives (FN): Buffered FRP clusters not intersected by any algorithm detection polygon.

The resulting aggregated classification metrics, evaluated against a total of 349 verified FRP-derived ground-truth fire events over the 11-day observational period, are analyzed below.

The quantitative metrics reported in Table 3 confirm the overall feasibility of utilizing the new MTG-FCI data for near real-time fire monitoring. The results demonstrate that the spectral and temporal resolutions of this dataset provide sufficient informational content to enable effective fire detection, yielding robust performance indicators across different algorithmic implementations.

In the Thresholding method, the data yields a high precision of 82.44% and a low False Alarm Rate (FAR) of 17.56%. This high reliability is direct consequence of the strict parametric constraints of the baseline thresholds, which successfully isolate prominent active fronts. However, this rigid setup restricts the extracted Recall to 48.29% due to the systematic omission of smaller or less intense events (FN=181), resulting in a cumulative $F_{1}$-score of 0.6090.

In the BLU-Net architecture, the system registers 254 True Positives, expanding the recall to 73.62% while maintaining a precision of 79.62% and a FAR of 20.38%. This balanced distribution between omission and commission errors results in a cumulative $F_{1}$-score of 0.7650.

Collectively, the quantitative indicators achieved by both configurations demonstrate the technical feasibility of the evaluated data source. However, it must be emphasized that referencing the performance validation exclusively against operational FRP products represents a highly conservative evaluation framework that introduces structural uncertainties directly affecting the False Positives (FP) and False Alarm Rate (FAR) reported in Table 3. While a strict multi-temporal verification using dNBR is bounded by the lack of perfect temporal coincidence, the empirical analysis indicates that when a post-event burn scar is identified within a restricted interval of 5 to 6 days and geographic constraints force the scar to be nested precisely within the detection polygon, the causal probability of a correct detection increases significantly. This spatial evidence strongly implies that a substantial portion of the 65 registered FPs for BLU-Net do not represent actual algorithmic failures, but rather real, small-scale or short-lived fire events omitted by the polar-orbiting FRP sensors due to temporal undersampling. Consequently, the actual operational FAR is inherently lower, and the precision higher, than the conservative statistical limits established by the strict FRP baseline.

To evaluate the metrics in Table 3, an error analysis was conducted on the 11-day dataset. For the Thresholding method, the fixed parametric triggers minimize false alarms (FP=36, FAR=17.56%) but increase omissions (FN=181, recall=48.29%), primarily failing to detect low-intensity or short-duration thermal signatures that do not reach the global activation threshold.

Conversely, the BLU-Net architecture reduces these omissions (FN=91, recall=73.62%), successfully identifying smaller sub-pixel hotspots due to its adaptive reconstruction framework. However, this higher sensitivity introduces an apparent increase in statistical commission errors (FP=65, FAR=20.38%). Crucially, this inflation does not purely reflect operational false alarms; while a subset of these instances stems from non-fire background variations (such as severe solar reflections or sharp thermal gradients) generating reconstruction residuals that exceed the combined binarization thresholds, our cross-validation with Sentinel-2 imagery confirms that a significant portion tracks real physical ignitions. Because these small-scale or transient outbreaks fall below the detection capabilities or overpass frequencies of polar-orbiting satellites, they are mathematically penalized as FPs under this strict reference baseline, thereby artificially inflating the statistical FAR beyond the model’s true operational error rate.

### 3.4. Temporal Advantage and Early Warning Potential

Comparing the detection times of MTG-FCI events with FRP observations demonstrates the advantage of geostationary sampling for early warning. On 5 August, the Vesuvius ignition was identified at 14:20 UTC, approximately 5 h before the first available FRP data (19:45 UTC). A similar gap was observed in Catania, where the fire was detected at 08:10 UTC, nearly 3 h before polar sensors recorded the first hotspots. Foggia presents a different case: MODIS Aqua (1 km resolution) recorded an earlier detection at 13:10 UTC, 30 min before the MTG-FCI identification. This gap is attributed to the trade-off between coarser spatial resolution and the statistical sensitivity thresholds of the unsupervised configurations. A low-magnitude thermal signature might appear in earlier FCI images, but it only stands out statistically as the fire grows to prevent false alarms.

**Figure 10:**
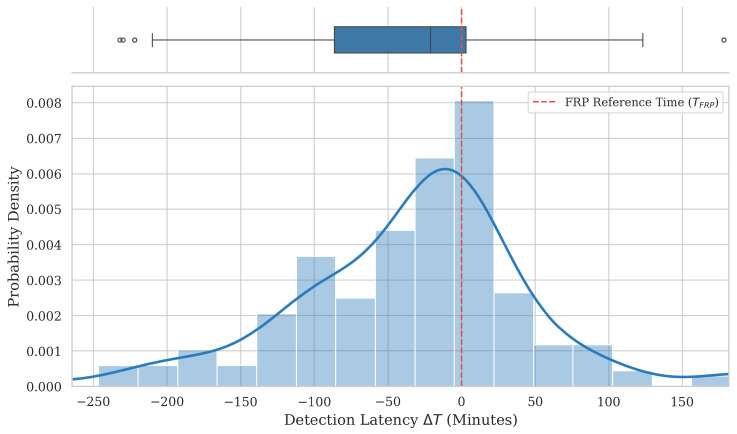
Empirical probability density distribution and boxplot breakdown of the detection latency (ΔT) computed over 254 synchronized fire events. The top panel illustrates the quartile distribution and extreme outliers, while the bottom panel presents the kernel density estimation (KDE) relative to the FRP reference baseline ($T_{\mathrm{FRP}}$).

To expand beyond these specific case studies, a comprehensive statistical evaluation was conducted across the subset of N=254 synchronized fire events successfully detected as True Positives, as illustrated in Figure 10. The temporal delta (ΔT) is computed by subtracting the BLU-Net detection time from the official FRP acquisition time; consequently, negative values represent an early detection advantage for the geostationary system, whereas positive values indicate a detection delay. The overall distribution confirms a predominant early warning tendency across the evaluation period, exhibiting a negative median delta of −21.00 min and a mean temporal delta μ=−37.16 min (σ=88.59 min). This left-skewed profile is driven by extreme lead-time outliers, reaching a maximum advance of −354.0 min (≈5.9 h), which highlights operational scenarios where geostationary sampling successfully bypasses the prolonged revisit intervals of polar-orbiting platforms.

The BLU-Net triggered active fire alerts prior to the official FRP validation in 67.32% of the tested occurrences (171 events, where ΔT<0 denotes early detection). Conversely, delayed detections (ΔT>0) accounted for 29.53% of the sample population (75 events), exhibiting a maximum delay of 210.0 min (≈3.5 h), while 3.15% (8 events) showed exact temporal synchronization (ΔT=0). These localized detection delays are primarily attributable to the conservative statistical constraints of the unsupervised algorithms implemented to mitigate false commissions, rather than an informational deficiency in the source data. These quantitative metrics confirm that the primary advantage of geostationary monitoring is alerting speed and temporal continuity rather than spatial precision, providing a critical operational window for rapid intervention.

### 3.5. Throughput Analysis and Operational Feasibility

The objective of this throughput analysis is to verify the operational feasibility of the MTG-FCI data stream in an NRT monitoring context, rather than to establish a performance competition between different algorithms. Given the sensor’s high revisit frequency (10 min for Full Disk or 2.5 min for RSS), the primary constraint is ensuring that any unsupervised detection logic can process the incoming data without accumulating lag. This prevents computational bottlenecks in the NRT pipeline, regardless of the underlying hardware architecture used (CPU vs. GPU).

**Figure 11:**
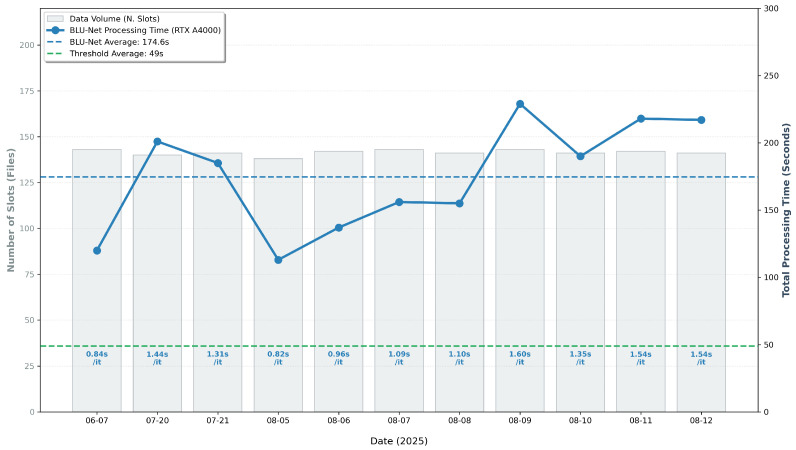
System throughput and latency analysis on NVIDIA RTX A4000 on BLU-Net and 12-core Intel(R) Xeon(R) E-2236 CPU @ 3.40GHz on Threshold method. Bars represent the daily data volume (number of slots), with internal labels indicating the average processing time per acquisition (s/it) in blue. Dashed lines provide a performance benchmark for the daily total execution time of the neural architecture (blue) compared to the deterministic threshold method on CPU (green).

Real-time monitoring requires processing the continuous data stream from the MTG-FCI sensor without accumulating lag. Benchmark tests performed on an NVIDIA RTX A4000 GPU show an average daily processing time of 174.6 s for a workload of approximately 140 slots. This throughput is roughly 500 times faster than the nominal satellite acquisition rate, providing an operational window for multi-regional monitoring or additional analysis layers. Specifically, per-slot inference times range between 0.82 s and 1.60 s. By comparison, the deterministic Threshold method on 12-core Intel(R) Xeon(R) E-2236 CPU @ 3.40GHz requires approximately 49 s per day (Figure 11). The two min difference introduced by the neural architecture is operationally insignificant relative to the 10 min acquisition cycle. Both methods maintain minimal alert latency and remain compatible with standard hardware requirements for functional centers.

### 3.6. Limitations

The main limitation of MTG-FCI data for fire detection lies in their spatial resolution, which is insufficient to track fire fronts or perform detailed perimeter mapping. Cloud cover can further hinder detection. Additional limitations arise from the detection methods themselves, which currently rely heavily on thresholding and are therefore sensitive to parameter selection.

Furthermore, the findings of this feasibility study are subject to constraints regarding geographic and temporal extrapolation. Since the evaluation is strictly calibrated on the 2025 Italian summer fire season, the generalizability of the algorithms to different ecosystems or winter fire dynamics remains to be verified. Additionally, a quantitative comparison with external geostationary AI algorithms or complex supervised architectures (such as Vision Transformers or deep CNNs) falls outside the scope of this feasibility study. Supervised models require large historical training datasets of annotated fire pixels, which are unavailable for a newly operational sensor like MTG-FCI. Furthermore, a direct performance benchmark against polar-orbiting products (MODIS/VIIRS) is constrained by the temporal sampling mismatch, as LEO satellites provide only intermittent overpasses. Consequently, MODIS and VIIRS products were utilized as validation references rather than direct algorithmic competitors, establishing a conservative baseline to verify the performance of the proposed unsupervised workflows. Additionally, while the BLU-Net model effectively learns to reconstruct static industrial sites and urban heat islands as part of the normal background state, exceptional high-intensity thermal events in these areas might still trigger false positives. Similarly, the fixed Thresholding method is susceptible to commission errors from solar reflections or intense ground heating during periods of extreme summer temperatures.

Specific operational failure modes are dictated by cloud coverage, geometric-solar illumination conditions, and surface-emissivity anomalies. Due to the absorption properties of infrared bands, fire anomalies are detectable only under clear-sky conditions; the signal is obscured when a cloud structure covers the burning area. However, a more nuanced failure case relates to cloud contamination during twilight periods (dawn and dusk). At low solar elevation angles, grazing incident sunlight triggers intense forward-scattering and multi-reflection effects precisely along the edges of high-altitude or convective cloud structures. These localized, high-contrast illumination spikes generate high reconstruction residuals in the BLU-Net model that exceed the learned background variance, occasionally triggering false positives.

Similarly, highly reflective and thermally responsive hot surfaces represent a known source of commission errors during peak summer hours. Anthropogenic structures, such as extensive solar photovoltaic plants, urban concrete roofs, and industrial complexes, undergo severe thermodynamic heating combined with specular solar reflection. Although our spatial buffering mitigates stable hotspots, extreme diurnal radiometric spikes in the mid-infrared spectrum can still mimic sub-pixel fire signatures.

Furthermore, seasonal environmental variability presents a constraint for unsupervised background modeling; rapid phenological transitions, such as intense summer droughts that sharply decrease soil moisture or localized agricultural harvesting that suddenly exposes bare soil, temporarily alter the surface emissivity. Because these rapid landscape variations deviate from the transitional baselines learned during the training phase (May and late September), they can artificially elevate the reconstruction noise floor, potentially masking low-intensity fires or causing ephemeral false alarms.

Lastly, despite the sub-pixel sensitivity of infrared channels, a lower boundary exists for the minimum fire surface area. If the extent or intensity of the ignition is too small relative to the geostationary pixel footprint, the thermal emission is diluted by the surrounding background radiance, preventing detection by both the autoencoder and the threshold filters.

## 4. Conclusions

In this study, we explored the feasibility of an unsupervised near real-time fire monitoring method using the new multispectral MTG-FCI data. The quantitative assessment shows that the algorithm triggers alerts prior to polar overpasses in 67.32% of the synchronized cases, yielding a median alert latency of −21.00 min. In extreme cases, when ignitions occur immediately after a polar satellite overpass, these localized lead times can extend up to approximately 6 h. This temporal delta does not represent the computational processing time of the software, but quantifies the early warning window enabled by geostationary sampling to cover polar revisit gaps. The proposed method also demonstrated the capacity to isolate persistent thermal anomalies in complex environments characterized by industrial zones or multiple concurrent ignitions, despite the spatial resolution limitations inherent to the sensor.

Future developments will first focus on a rigorous, systematic sensitivity analysis of the operational thresholds to optimize the balance between detection rates and false commissions. Concurrently, we plan to integrate additional spectral bands and spatio-temporal architectures to leverage the 2.5-min Rapid Scanning Service (RSS). This high sampling rate is expected to improve the validation of transient thermal anomalies and reduce false positives by tracking the temporal evolution of the radiative signal. Operationally, the ingestion of the RSS stream requires optimizing the processing pipeline to ensure execution cycles are completed within the 150 s latency window.

While this research represents a preliminary study, the empirical results confirm the technical feasibility of the evaluated MTG-FCI data source for early fire detection. The capability to reduce the temporal gap between ignition and the first satellite alert provides an operational window for containment operations before the first polar satellite overpass. Ultimately, integrating these unsupervised workflows into operational monitoring networks demonstrates that geostationary satellite resources can serve as an effective component of automated decision support systems for early warning.

## Figures and Tables

**Table 1:** MTG-FCI spectral bands used in this study.

FCI Channel	Central λ (μm)	Spectral Width Δλ (μm)	Resolution (km)	Quantity
NIR1.6	1.61	0.05	1.0	Reflectance (%)
NIR2.2	2.25	0.05	1.0	Reflectance (%)
MWIR	3.80	0.4	2.0	BT (K)
TIR	10.50	0.7	2.0	BT (K)

**Table 2:** Sensitivity analysis of the architectural bottleneck dimension (*d*) and its impact on reconstruction metrics and downstream unsupervised fire detection performance.

Bottleneck Size (*d*)	Best Training Loss	Mean Training Residual	Sensitivity (TPR%)	FPR (%)	F1-Score (%)
1	$6.1\times 10^{-5}$	$7.9\times 10^{-3}$	13.0–27.0%	14.82%	11.2–21.4%
2	$5.0\times 10^{-5}$	$2.4\times 10^{-3}$	34.0–69.0%	5.16%	36.8–62.5%
4	$44.5\times 10^{-5}$	$8.5\times 10^{-3}$	82.0–87.0%	0.94%	78.5–83.2%
6	$4.8\times 10^{-5}$	$5.1\times 10^{-3}$	55.0–68.0%	0.52%	61.2–71.8%
8	$5.4\times 10^{-5}$	$5.7\times 10^{-3}$	41.0–53.0%	0.31%	49.3–58.1%
16	$4.6\times 10^{-5}$	$3.1\times 10^{-3}$	9.0–27.0%	0.05%	14.1–34.6%

**Table 3:** Quantitative performance assessment of the Thresholding method and the BLU-Net algorithm aggregated over the 11-day evaluation period.

Algorithm	TP	FP	FN	Precision (%)	Recall (%)	$F_{1}$-Score	False Alarm Rate (FAR) (%)
Thresholding method	169	36	181	82.44	48.29	0.6090	17.56
BLU-Net	254	65	91	79.62	73.62	0.7650	20.38

## Data Availability

The MTG-FCI data analyzed in this study were received and archived locally via a dedicated EUMETCast High-Volume Service 3 (HVS-3) reception station installed at ISPRA. The raw data streams are also publicly available through the EUMETSAT Data Store (https://data.eumetsat.int). Sentinel-2 imagery was accessed via the Copernicus Data Space Ecosystem (https://dataspace.copernicus.eu), accessed on 21 October 2025. Fire Radiative Power (FRP) data were aggregated into a dedicated geodatabase from NASA FIRMS NRT products, including MODIS (Collection 6.1) and VIIRS (Collection 2) from the Suomi-NPP, NOAA-20, and NOAA-21 platforms (https://firms.modaps.eosdis.nasa.gov). Sentinel-3 SLSTR data (product EO:EUM:DAT:0417) are accessible via EUMDAC. Fire perimeter data were obtained from the European Forest Fire Information System (EFFIS, https://effis.jrc.ec.europa.eu).

## Abbreviations

The following abbreviations are used in this manuscript:

BT	Brightness Temperature
CAE	Convolutional Autoencoder
CNN	Convolutional Neural Network
dNBR	differenced Normalized Burn Ratio
EFFIS	European Forest Fire Information System
FAR	False Alarm Rate
FCI	Flexible Combined Imager
FDHSI	Full Disc High Spectral Resolution Imagery
FP	False Positive
FRP	Fire Radiative Power
GEO	Geostationary
HVS-3	High-Volume Service 3
IR	Infrared
ISPRA	Italian Institute for Environmental Protection and Research
JRC	Joint Research Centre
LEO	Low Earth Orbit
MODIS	Moderate-Resolution Imaging Spectroradiometer
MSG	Meteosat Second Generation
MSI	MultiSpectral Instrument
MTG	Meteosat Third Generation
MWIR	Medium-Wave Infrared
NIR	Near-Infrared
NRT	near-real-time
ReLU	Rectified Linear Unit
RSS	Rapid Scanning Service
SEVIRI	Spinning Enhanced Visible and Infrared Imager
SLSTR	Sea and Land Surface Temperature Radiometer
TIR	Thermal Infrared
TOA	Top of Atmosphere
U-Net	U-shaped Convolutional Neural Network
UMAP	Uniform Manifold Approximation and Projection
VIIRS	Visible Infrared Imaging Radiometer Suite
VIS	Visible
